# Exploration of novel bioactive compounds from the microbiome of fish and shellfish as an alternative to replace antibiotic drugs in aquaculture farming

**DOI:** 10.1017/gmb.2025.6

**Published:** 2025-05-14

**Authors:** Arvind Diwan, Sanjay Harke, Archana N. Panche

**Affiliations:** Institute of Biosciences and Technology, Mahatma Gandhi Mission (MGM) University, Aurangabad, 431003, Maharashtra, India

**Keywords:** gut microbiome, bioactive compounds, antibiotics, aquaculture, therapies

## Abstract

The use of antibiotics in fish and shrimp aquaculture all over the world was found to be only partially successful in preventing infectious diseases. However, their overuse has resulted in the contamination of closed aquatic ecosystems, reduced antibiotic resistance in organisms that fight infectious diseases, and compromised the effectiveness of various antibiotic medications in controlling diseases. Excessive use of antibiotics damages aquaculture species and impacts human health, also rendering the most potent antibiotics increasingly ineffective, with limited alternatives. Therefore, intensive research efforts have been made to replace antibiotics with other protocols and methods like vaccines, phage therapy, quorum quenching technology, probiotics, prebiotics, chicken egg yolk antibody (IgY), and plant therapy,” etc. Though all these methods have great potential, many of them are still in the experimental stage, except for fish vaccines. All these alternative technologies need to be carefully standardized and evaluated before implementation. In recent times, after realizing the importance of the gut microbiome community in maintaining the health of animals, efforts have been made to use the microbiome strains for the prevention of pathogenic bacterial and viral infections. Now it has been experimentally proven that animals should possess a healthy microbiome community in their gut tract to strengthen the immune system and prevent the entry of harmful pathogens. Investigations are now being carried out on the derivation of various bioactive compounds from the gut microbiome strains and their structural profile and functionality using the molecular tools of metagenomics and bioinformatics. Such newly discovered compounds from microbiomes can be used as potential alternatives to replace antibiotic drugs in the aquaculture industry. These alternatives are likely to emerge as breakthroughs in animal health management and farming, with effects on cost efficiency, species health, productivity, and yield enhancement. Therefore, introducing new micro-innovative technologies into an overall health management plan will be highly beneficial.

## Introduction

The gut microbiomes in most organisms are a rich source of bioactive compounds that have the potential to be developed into future antibiotics (O’Neill, 2014). These compounds include antimicrobial peptides, bacteriocins, and other small bioactive molecules that can interact with other pathogenic bacteria to inhibit or modify their growth and colonization (Tortorella et al., 2018). There are billions of microbes present in the body that have coevolved with organisms and play a pivotal role in controlling many physiological activities, including the strengthening of the immune system. The emerging resistance to known antibiotics for preventing the recurrence of diseases in aquaculture farming and also for the need for new and green antibiotics has led to the discovery of new bioactive compounds from microbiome strains. The microbiome species produce several bioactive compounds in response to either pathogenic bacteria and viruses or other environmental stresses. All such bioactive compounds produced have significant functional properties like antimicrobials and antibiotics and can be used for therapeutic purposes to mitigate infectious diseases in cultivable organisms. In the early 1960 an antibiotic like penicillin was discovered by screening 19 microbiome species, and later several novel bioactive compounds and antimicrobial peptides were isolated from the microbiomes of soil and marine, and other diverse habitats (Moore and Gerwick, 2012; Lam and Crawford, 2018). At present, several bioactive compounds are being discovered using a combination of genome mining, activation of silent biosynthetic pathways, and metagenomic analysis of a variety of ecosystems (Challinor and Bode, 2015; Garcia-Gutierrez et al., 2018). Even small biomolecules, peptides, and secondary metabolites are also being discovered from the microbiome species, and such compounds are obtained mostly from the commensal microbiomes and through microbe–host interactions. To understand the host-microbiome interaction and identify potential antimicrobial compounds focus of research is now given to large-scale genome sequencing of isolates of body parts of the cultivable organisms. Metagenomics and meta-transcriptome data collected from different commensal microbiomes were analyzed using bioinformatics applications to understand the functional aspects of molecules (Diwan et al., 2023, 2024).

In aquaculture farming, the surge of antimicrobial resistance infections and the concurrent increase in the usage of antibiotic drugs and other chemicals have jeopardized the healthcare system in cultivable organisms. This has created an unhealthy aquatic environment, creating opportunities for the entry of new pathogens. There is a need to build a robust healthcare system naturally, without using any drugs among the cultivable organisms, so that incidences of mass mortality and losses can be avoided. Therefore, the search for alternate antimicrobial agents has become a necessity. In recent years, emphasis has now been given to searching for natural products as sources of therapeutic agents, with antimicrobials being one of the most compelling biomolecules. Unlike microbial-originated antibiotics, plant-based antimicrobials have been extensively explored and with varied applications in medicine, veterinary, agriculture, and biotechnology. The microbiomes located in the gut system of animals have been recognized as producers of bioactive compounds with antibacterial, antifungal, and cytotoxic bioactivity (Xu et al., 2015; El-Demerdash et al., 2018; Karpinski, 2019; Elissawy et al., 2021). Due to their distinctive biological properties, researchers have recently identified microbes as untapped reservoirs for novel antimicrobial agents (Swift et al., 2021). Specifically, the invention of state-of-the-art molecular biology, genetic, genomic, and computational tools has facilitated the mining of microbial structural systems to enhance drug discovery (Moir et al., 1999; Schnappinger, 2015; Maghembe et al., 2020).

The microbiome present in the gut system of fish and shellfish is ubiquitous, diverse community of organisms broadly categorized into viruses, bacteria, archaea, fungi, and protists. Among these microbiome communities, bacteria and fungi have been explored as potential sources of novel antimicrobial bioactive compounds. There are reports regarding the isolation of several peptide compounds (mathiapeptide, destotamide, Marfomycins, spirotetronates abyssomycin, and Lobophorin) from the bacteria including *staphylococcus aureus*, *Micrococcus luteus, Bacillus subtilis, Enterococcus faecalis* having the properties of antimicrobial compounds. Several researchers have conducted in vivo and in vitro studies to show that the bioactive compounds extracted from Cyanobacteria, yeast, and microalgae have antibacterial functions. Such discoveries of antimicrobial compounds from microbiome species have inspired several workers to produce synthetic antimicrobials from natural products to overcome antibiotic resistance (Mitcheltree et al., 2021). Due to the emergence of diverse strains of microbiomes having antimicrobial properties and the advent of modern scientific tools for analysis, there is a large potential and opportunities to enhance the bioprospecting of new antimicrobial compounds. Therefore, the main objectives of this review are to investigate and report the novel antimicrobial compounds from the gut microbiome species of fish and shellfish of aquaculture importance and further explore their potential use for therapeutic purposes, replacing antibiotic drugs.

## Antibacterial compounds from the bacterial community

Since the ban on antibiotics in aquaculture farming, emphasis is now being given to searching for natural products as sources of therapeutic agents to prevent the recurring spread of diseases in fish and shellfish during their captive culture. The plant-based anti-microbial has been extensively explored, and its therapeutic use in medicine, veterinary, agriculture, and biotechnology has been well-proved. However, anti-microbial compounds from microbiomes of fish and shellfish have not been explored much, and extensive research is warranted in this niche area of science (Diwan et al., 2023). The gut microbiome species present in fish and shellfish have been recognized as the producers of several novel bioactive compounds with antibacterial, antifungal, and cytotoxic functional properties (Xu et al., 2015; El-Demerdash et al., 2018; Karpinski, 2019; Elissawy et al., 2021). They also produce functionally rich secondary metabolites, which enable them to survive in varied environmental conditions. Several reports in the recent past mention that microbes are unexplored reservoirs of novel antimicrobial agents due to their distinctive biological properties (Swift et al., 2021). Because of the advancement in analytical molecular tools, the innovative inventions in molecular genomics and genetics, and in-silico technology have facilitated the mining of microbial structural profiles to enhance drug discovery research (Moir et al., 1999; Schnappinger, 2015; Maghembe et al., 2020). The microbiome community in such cultivable aquatic organisms is ubiquitous, diverse in composition, and broadly categorized into viruses, bacteria, archaea, fungi, and protists. Predominantly, bacteria and fungi are explored as potential sources of novel antimicrobial agents. Sanchez et al. (2012), while working on the marine fish gut microbiome, reported that several natural bioactive compounds produced by the gut microbiomes possess significant functional properties.

The microbiome belongs to the group Actinobacteria, including *Rhodococcus*, *Microbacterium*, and *Micromonospora* species, which are present in the digestive tract of six varieties of fish predominantly, and produces a novel lipid compound, viz., sebastenoic acid. Many researchers have reported that Sebastenoic acid has antibacterial properties that protect organisms against bacterial infection, particularly from strains like *Staphylococcus aureus*, *Bacillus subtilis, Enterococcus faecium*, and *Vibrio mimicus*. Diwan et al. (2021) in their review paper mentioned about 1000 new bioactive compounds produced by the microbiome species associated with several marine invertebrates. As the microbiome species produce several vital bioactive compounds having the properties of antimicrobials, a lot of research work has been done on genome sequencing of these microbiome communities (Van Trindade et al., 2015). It has been further reported that 37 secondary metabolites have been discovered from the gene clusters of the fish gut microbiome species like *Streptomyces avermitilis*, having the properties of antimicrobials. The strains of firmicutes and proteobacteria from the gut of fish have been shown to contain active biological ingredients having functional properties against Gram-positive and Gram-negative pathogenic bacteria. Several peptides, alkaloids, and sesquiterpenes have been isolated from the different bacterial species, having the properties of antimicrobials (Tortorella et al., 2018). Furthermore, in vivo and in vitro assays have also demonstrated the anti-infective potential of other microbial products extracted from cyanobacteria, microalgae, and yeast (Rojas et al., 2020; Alsenani et al., 2020). Several workers have reported that the lactic acid bacteria found in the gut of fish produce bacteriocin compounds that are effective against pathogenic bacteria (Tenea et al., 2016). Similarly, bacteriocin produced by *Lactobacillus pentosus* has been found to arrest the proliferation of certain pathogenic bacteria like *E. coli, Pseudomonas aeruginosa, E. faecalis, K. pneumoniae*, and *Lactobacillus curvatus* (Todorov and Dicks, 2007). Bonnie Waycott (2023) mentioned that the bacteriocin produced by the *lactobacillus* sp. can be used as an antibiotic alternative. It was confirmed by the application of bacteriocin in shrimp farming. Bacteriocins are low molecular weight polypeptides synthesized in ribosomes and contain 20–60 amino acid residues. Since the discovery of different forms of bacteriocin from microbial species and their antimicrobial properties, several workers have taken an interest in this product and worked out their mechanisms related to antimicrobials (Oscáriz and Pisabarro, 2001; Cotter et al., 2005; Güllüce et al., 2013; Herzog et al., 2020; Jiang et al., 2020; Hernández-González et al., 2021; Thakur et al., 2021). These compounds have shown various biological activities against microorganisms, biofilm, tumors, and oxidation. Several biopharma industries are now looking for such new microbial products from the microbiome species of both fish and shellfish, and manufacturing these products under different commercial names for their use in aquaculture farming. To promote the prospects of such microbial products in these industries, there is an urgent need for intensive research on fish and shellfish microbiomes, particularly in genomics and metagenomics areas. The functional aspects of the gut microbiome are an important issue for future investigation, and therefore, in-depth knowledge of functional metagenomics is very much essential. Table 1 summarises fish and shellfish microbiomes producing bioactive compounds for their potential use in the development of aqua-bio industries. It has been reported that *Actinobacteria* found in the gut of several marine fish produce many bioactive compounds, including the most important one i.e., bacteriocin. Another compound called anthramycin which has the property of an antibiotic, is produced by actinomycete bacteria, which works against *Bacillus anthracis*, a pathogenic bacterium.

**Table 1.** Bioactive compounds derived from the gut microbiomes of finfish and shellfish

Finfish	Gut microbiomes	Bioactive compounds	Possible functions	Reference
	Microbes (*Atlantic Salmon*)	Antibiosis	Antibacterial	Liu et al. (2010) andCaruffo et al. (2015)
	Microbes Seabass	Beta-lactamases	Antibacterial	Xia et al. (2014) and Ringø and Holzapfel (2000)
Fish	Microbes	Lipoprotein Lipopolysaccharides, peptidoglycan, bacterial flagellin	Recognition of immune receptors	Pietretti and Wiegertjes (2014) and
Freshwater carp	*Clostridium* sp.	Lactic and phosphoric acid	Strengthening of the immune system	Li et al. (2017)
Shrimp	*Bacillus* sp.	Anti-bacterial peptides	Prevent entry of pathogens	Proespraiwong et al. (2023)
Fish	Luminal bacteria	Mucins, enzymes, piscidins, defensins	Anti-microbial	Zou et al. (2007), Silphaduang et al. (2006) and Hormannsperger and Haller (2011)
Shrimp	*Vibrio* sp.	Chitinolytic enzymes	Decalcifying effects	Jayasree et al. (2006)
Fish	Actinobacteria	Sebastenoic acid	antimicrobial	Sanchez et al. (2012) and Tyagi et al. (2019)
Fish	*Cetobacterium* sp.	Vitamin B12	Strengthening immune system	Larsen et al. (2014), Lyons et al. (2015) and Gaulke et al. (2016)
Shrimp	Photobacterium Aeromonads, Bacillus, Vibrio	Chitinase enzyme	Degradation of chitin	Itoi et al. (2006)
Fish	*Clostridium* species	Fatty acids, Vitamins Enzymes-Acid and Alkaline phosphatases	Strengthening immune system	Ramirez and Dixon (2003)
Fish	Actinomycetales Firmicutes Proteobacteria sp.	Antibacterial compounds	Anti-bacterial	Van Trindade et al. (Van Trindade et al., 2015)
Shrimp	*Streptomyces avermitilis*	Secondary metabolites	Anti-bacterial	Van Trindade et al. (2015)
Fish	*Cetobacterium* sp., Fusobacteria	Vitamin B–12-like compounds	Vitamin B12	Tsuchiya et al. (2008), Roeselers et al. (2011), Ye et al. (2014), Liu et al. (2016) and Eichmiller et al. (2016)
Fish	*Lactobacillus*, *E. coli*, Bacteroides	Digestive enzymes (b-glucanases, carbohydrases, cellulases, chitinases)	Cellulose digestion enzyme products	Ray et al. (2012)
Fish/Shrimp	*Clostridium* group	Enzymes for collagen digestion Propionate compounds, short chain fatty acids, butyrate, collagen-degrading enzymes, acid and alkaline phosphatase enzymes	Enzyme production for collagen degradation Growth promoting stimulants Probiotics	Baldo et al. (2015), Eichmiller et al. (2016) and Ramirez and Dixon (2003)
Fish Shrimp	Luminous and nonluminous photobacteria	Enzyme chitinases	Chitin digestive enzymes	Itoi et al. (2006), Ward et al. (2009) and Smriga et al. (2010)
Fish/Shrimp	Proteobacteria	Degrading enzymes for cellulose, hemicellulose, chitin, pectin, starch, etc	Enzymes in the form of probiotics supplements	Tyagi et al. (2019)
Fish	*Vibrio* sp.	Hydrolytic enzymes, amylases, lipases, cellulases, and chitinases	Digestive enzymes products	Itoi et al. (2006) and Sugita and Ito (2006)
Fish/Shrimp	*Lactobacillus*, *Enterococcus*, *Bacillus*, *Aeromonas*, *Alteromonas*, *Arthrobacter*, *Bifidobacterium*, *Clostridium*, *Microbacterium*, *Paenibacillus*, *Phaeobacter*, *Pseudoalteromonas*, *Pseudomonas*, *Rhodosporidium*, *Roseobacter*, *Streptomyces* and *Vibrio*	Growth and immunostimulants bioactive compounds	Probiotics	Ringø (2020)
Shrimp	*Aliivibrio* bacteria *Lactobacillus*	Bacteriocins	Probiotics	Bonnie Waycott (2023)

Anthramycin is also found to be produced by the gut bacterium *Streptomyces* from marine environments, particularly in higher organisms 65 (Valliappan et al., 2014). Actinobacteria from marine fish have been the focus of several researchers as this group of microbiomes produces many antibacterial compounds (Vignesh et al., 2019; Vadivel et al., 2021). The antimicrobial activity of Actinobacteria against pathogens like *Salmonella enterica*, *S. aureus, E. coli*, and *Streptomyces bacterium, working as* antimicrobial, antifungal, and quorum-sensing inhibitory activity, has been well-established in the recent past.

**Figure 1.**
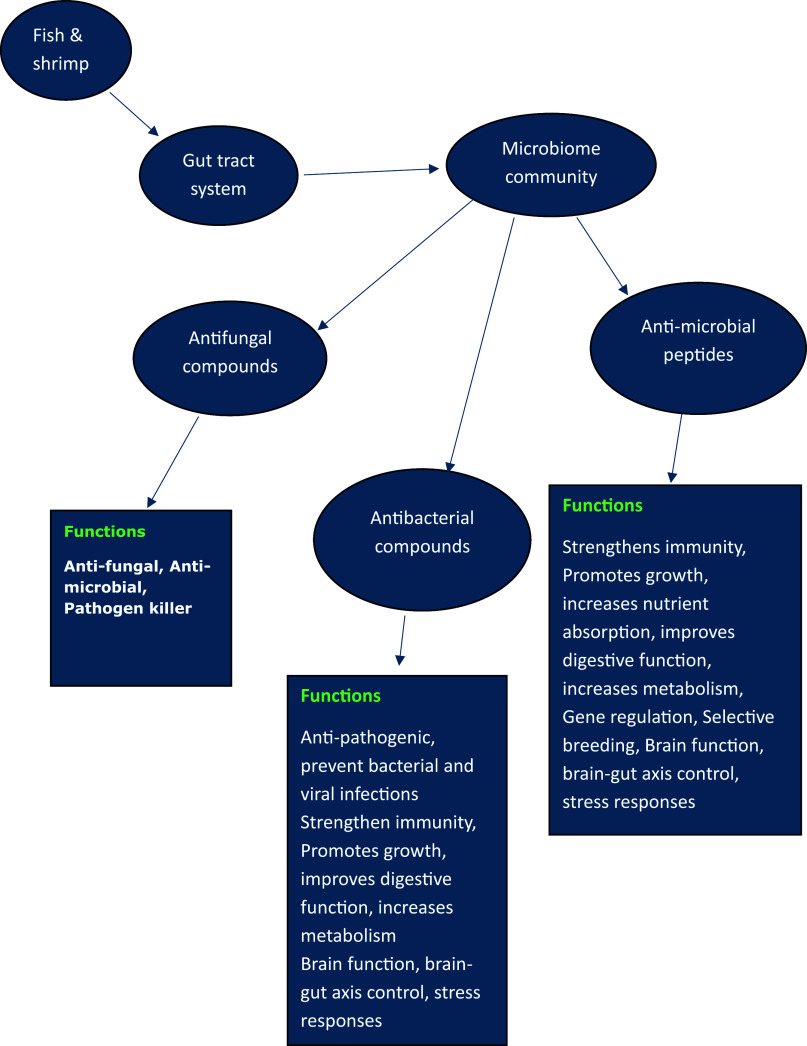
Illustrates the functional aspects of different microbial compounds produced by the gut microbiome community in fish and shrimp.

After realizing the importance of fish gut microbiomes as the best resources for producing novel bioactive compounds, one of the challenges researchers faced was how to culture these microbiome strains under lab conditions for their large-scale production and utilization in aquaculture to replace antibiotic drugs. It was also felt that the structure and functional properties of the bioactive compounds they produce in laboratory culture systems should not be altered as several environmental factors including the temperature, PH, and dissolved O2 concentration may affect not only the diversity of microbiome composition but also the nature of the bioactive compounds (Abe and Nakazawa, 1994; Lim et al., 1994; Knappe et al., 2008; Saalim et al., 2020). It is reported that in lab culture of such microbiome strains, often, modified or selective media are often used with long incubation times of days, or even weeks, for antimicrobial production to occur. Sanchez et al. (2012), while working on the fish gut microbiome, used selective media for the lab culture of *Actinobacteria* to produce bioactive enzymes. For s*treptomyces* culture in the lab, Vadivel et al. (2021) mentioned that by substituting carbon, nitrogen, and salt sources as well as altering the pH, there was a drastic change in the antimicrobial properties of *streptomyces.* Uniacke-Lowe et al. (2024) while working on the gut microbiome of fish as a source of bacteriocins, reported that several environmental factors such as high salinity, hydrostatic pressure and a range of environmental temperatures all have an impact on the structural and functional diversity of marine antimicrobial molecules produced in the gut microbiomes, including bacteriocins. Further, it is mentioned that the characterization of the diversity of microbiome composition in such variable environments and the nature of the bioactive compounds they produce are still future challenges that require a lot of research investigations. In such challenges, advanced molecular tools like metagenomics, bioinformatics, and next-generation sequencing technologies will greatly support resolving the fish gut microbiome’s taxonomic diversity and identifying the microbiome ingredients with their functional aspects. Efforts have been made to summarise different antimicrobial compounds produced by fish and shellfish microbiomes in Figure 1.

## Antifungal compounds from the microbiomes

The fungal diseases in fish and shellfish are common and, if left untreated, can cause a secondary infection, leading to septicemia, fin rot, or dropsy. Further, it can severely damage the fish population and lead to heavy mortality in aquaculture farming systems. The fungus infection is a disease that affects mainly the skin and gills of fish and shellfish, and spores of the fungus cause fish fungus (Brown et al., 2012). Many different species of fungus can infect fish, and these include Saprolegnia, Achylia, and Fusarium. To prevent the spread of fungal diseases, standard protocols are available in the literature, including the use of antibiotics in severe cases of fungal infections. To replace antibiotics, research is being focused on using microbiome therapies or new antifungal drugs derived from the marine invertebrate (Zhang et al., 2020). There are reports that the killer fungus, *Candida auris* is spreading in healthcare facilities worldwide, and cautious threat alerts are being issued from time to time by the Centers for Disease Control and Prevention, USA (Meis and Voss, 2019; Zhang et al., 2020) for controlling the spread of the fungus. However, preventing the spread of this fungus using multiple drugs has been tried by several researchers, but with limited benefits. In recent years because of the invention of molecular tools like liquid chromatography-mass spectrometry (LC–MS)–based metabolomics, and antimicrobial activity screening of metabolomic arrays from the microbiome isolates of marine animals (Hou et al., 2012; Chanana et al., 2017), emphasis is now being given to finding out new natural bioactive compounds from the gut microbiome strains which are effective in preventing fungus infection.

Zhang et al. (2020), while working on the discovery of a new anti-fungal drug from the marine microbiome, *Micromonospora* sp, found that turbinmicin is a promising antifungal bioactive compound. in vitro and in vivo, this compound exhibited powerful antifungal activity against emerging multidrug-resistant human fungal pathogens, including *C. auris.* Turbinmicin belongs to a small group of polyketides. It has been reported that this compound is highly antimicrobial and works effectively against *C. auris*, and *Aspergillus fumigatus.* This prominent functional antimicrobial property supports the compound’s development for future clinical use. Turbinmicin, when it was tested with different concentrations and doses on other fungal species like *Candida albicans*, *C. glabrata*, *C. tropicalis*, *Aspergillus fumigatus*, *Fusarium* spp., *Scedosporium* spp., and *Rhizopus* spp., was found to be very effective. Kingwell (2021), while working on the marine microbiome and antifungal properties, made a similar observation about the compound turbinmicin and its significant role in controlling the fungus infection caused by *C. albicans.* Another microbiome species from marine habitats *Micromonospora*, has been identified with the production of antifungal compounds. These species produce a secondary metabolite called spartanamycin, which is active against *C. albicans, Aspergillus Cladosporium*, and *cryptococcus* sp. (Kerr, 1999; Boumehira et al., 2016). Boumehira et al. (2016) also reported that Micromonospora neiheumicin produces an antifungal compound, neiheumicin, which works against *Saccharomyces cerevisiae.* Some workers have reported that bacilli species are good sources of producing several antifungal compounds, like iturin, bacillomycin, mycosubtilin, and mojavensin (Kerr, 1999; Dunlap et al., 2019). According to Kerr (1999), anti-microbial compounds like azoxybacilin, bacereutin, cispentacin, and mycocerein are found to be produced by *Bacillus cereus*, and all these compounds work against Aspergillus species, Saccharomyces spp., Candida albicans, and other fungi. Chernin et al. (1996) reported that *Enterobacter s*p. produces a compound called herbicolins, which has been found to be active against yeasts and filamentous fungi. Similarly*, Pseudomonas sp.* has been reported to produce antimicrobial compounds, pseudomycin, caryoynencins, and cyclic hydroxamic acid (Vincent et al., 1991; Yamaguchi et al., 1995; Kerr, 1999).

## Antimicrobial peptides from microbiomes

Several workers have reported that the microbiomes produce various antimicrobial peptides and play a significant role in the host defense systems (Wang et al., 2019). These antimicrobial peptides are small-sized protein compounds consisting of large numbers of lysine and arginine residues, which are cationic. These compounds positively charged cationic properties enable them to react with microbial membranes of infectious bacteria that are negatively charged (Narayana and Chen, 2015). In aquaculture farming, infection with bacteria and viruses is so common, and every time, there is no alternative technology except to use antibiotic drugs to control such infectious diseases. However, after discovering antimicrobial peptides from microbiome species and their vital role in controlling diseases, much attention is now paid to replacing antibiotic drugs with antimicrobial peptides (Danquah et al., 2022). Recently, antimicrobial peptides have shown excellent antibacterial activity against harmful pathogenic microorganisms by acting on multiple target points (Huerta-Cantillo and Navarro-García, 2016; da Cunha et al., 2017). A few reports also indicated that antimicrobial peptides act as antifungal, antiviral, antiparasitic, and immunomodulatory agents (Wang et al., 2019). Even common pathogenic bacteria like *Acinetobacter baumannii, Listeria monocytogenes*, *E. coli*, and *Vibrio parahaemolyticus* are affected and controlled by antimicrobial peptides. The antimicrobial peptides like nisin, cecropins, and defensins have shown excellent results in preventing infection caused by Gram-positive and Gram-negative bacteria. 104 Though applications of antimicrobial peptides are diverse, their potential use has good scope not only in the aquaculture industry but also in human medicines, the food industry, agriculture sector; however, their production at the industrial scale is low. One reason speculated for low production is that the antimicrobial peptides are susceptible to proteolytic degradation due to the L-amino acids in them (da Cunha et al., 2017). Hence, genetic engineering tools are now being used to increase the production of antimicrobial peptides with better functional properties.

## Therapeutic applications

It is well-established that the commensal microbiome community available in the gut tract of fish and shellfish plays a significant role in building strong immunity to manage the prevention of infectious diseases and keep the animal’s body healthy. Therefore, in aquaculture farming, several researchers have focused on investigating the possible role of antimicrobial compounds produced by the microbiome species in fish gut tracts in controlling bacterial and viral diseases. Many investigations in the recent past have also emphasized their research programs on interactive mechanisms between commensal microbiomes and pathogenic bacteria and viruses (Diwan et al., 2023). Zhang et al. (2010) and Salinas et al. (2011) discovered a compound called “immunoglobulin” while working on fish microbiomes, and this compound they derived from the interaction between commensal and pathogenic bacteria present in mucosal layers of the skin and other tissues. Further, these authors have mentioned whether the mechanism involved in immunoglobulin production by the microbes present in the gut tract and skin tissues in response to pathogenic bacteria is similar to the mechanisms that also exist for the gill tissues. Zhang et al. (2010) reported the presence of different types of immunoglobulins in the gill tissues of rainbow trout, performing the function of defense, and from these findings, it was speculated that the possibility of developing a cost-effective vaccine from microbiomes for controlling infectious diseases (Rawls et al., 2004). Many findings suggest that the genes in the fish body that control different physiological activities in the digestive tract, including functions like immunity, nutrition, and metabolism, are also governed by the gut microbiomes. Hence, there is an urgent need to identify these microbiomes, their ingredient molecules, functions, and their interaction with the host microbiome. Perez et al. (2010) found that the lymphoid tissues that are associated with the gastrointestinal tract can identify the pathogenic bacteria in the gut system, and at the same time, the commensal microbiomes regulate the immune system in fish. From some of these observations, it was inferred that the microbiome present in the intestinal mucosal layers produces many antimicrobial compounds mucins, enzymes piscidins, and defensins in response to pathogenic bacteria, and this sort of reaction is the first line of defense provided in fish and the second line of defense is the lymphoid tissues which produce many immunoglobulins as mentioned earlier (Silphaduang et al., 2006; Zou et al., 2007). It has been observed that the continuous use of antibiotic drugs, vaccines, and other chemotherapeutic methods to control the frequent occurrence of bacterial and viral diseases in aquaculture farming has offered limited solutions and allowed the entry of more infectious new pathogens. Therefore, new methods and protocols based on natural compounds are necessary for the purpose. Becattini et al. (2016) while working on fish microbiomes reported that the microbe, *Clostridium butyricum* produces short-chain fatty acids that help the animal not only provide energy for the regeneration and repair of epithelial cells of the gut tract but also to reduce the pH of intestinal fluid, promoting the growth of bacteria, and preventing the entry of invasive pathogens. There are also reports that *C. butyricum* produces bacteriocin and lipoteichoic acid that act as antibacterial compounds (Gao et al., 2011, 2013; Junghare et al., 2012). Applications of *C. butyricum* in preventing *Vibrio harveyi* attack in freshwater prawn Macrobrachium rosenbergii have been reported by Sumon et al. (2018). Duan et al. (2017) also observed that the presence of *C. butyricum* in the shrimp is advantageous in preventing bacterial and viral infection, strengthening the immune system, and managing thermal stress. The production of antibacterial compounds like crustin, prophenoloxidase enzymes, lysozymes, and glucan-binding proteins from *C. butyricum* in the gut tract of shrimp and their role in controlling infectious diseases have been reported by Miandare et al. (2017) and Chen et al. (2016). While investing in the health benefits of the bacterium *C. butyricum* and the butyrate in fish and shellfish, Tran et al. (2023) in their paper mentioned that this bacterium can enhance the growth performance of cultured animals. This is because *C. butyricum* facilitates the structural modification of surface layers in the intestine and the activity of digestive enzymes, as well as modulating gut microbiota, with an increase in the commensal bacteria and a decrease in the population of infectious pathogens. Having realized the importance of *C. butyricum*, it is necessary to prioritize further research on this bacterium’s genomic and metagenomics profile so that such studies will help us in the larger production of antibacterial compounds for their wide application in controlling diseases. Similar research work must be planned to investigate more gut microbiome species that are involved in antibacterial functions.

Though a considerable amount of information is now available covering several aspects of the gut microbiomes in fish and shellfish, research on the functional aspects of microbial species at the genomic level is lacking. Several reports indicated that the gut microbiome plays an important role in maintaining the proper growth and health of the animals. In earlier years, they indicated that the gut microbiome in fish and shellfish produces various digestive enzymes. However, some enzymes like β-glucanases, carbohydrases, cellulases, and chitinases fish do not produce particularly herbivorous and detritivorous species. In such animals, the digestion of cellulose is taken over by the microbiome species present in the gut system at that time (Ray et al., 2012). Tsuchiya et al. (2008) reported that microbiome species like *Cetobacterium somerae* present in the gut tract produce an abundant amount of vitamin B12. Findings of *Cetobacterium* producing vitamin B12 have been supported by Romero et al. (2014), who also mentioned that this bacterium produces vitamin B12 in fish like Nile tilapia and common carp *Cyprinus carpio*, which have no dietary vitamin B12 requirement. However, fish species like channel catfish *Ictalurus punctatus*, and Japanese eel *Anguilla japonica*, in which *Cetobacterium* is not commonly present in their gut system, require dietary vitamin B12 from external sources. Some other workers reported that the production of various short-chain fatty acids occurs when gut tract microbes are involved in the digestion of dietary fiber, particularly in herbivorous fish species (Mountfort et al., 2002).

Several reports have described the functional aspects of short-chain fatty acids in many fish, emphasizing that the presence of short-chain fatty acids can make the environment non-conducive to some potential pathogens and increase the solubility of minerals, making them more easily absorbed (Mountfort et al., 2002; Merrifield and Rodiles, 2015). Certain findings have confirmed that the fishes cannot digest food containing cellulose and degrade and eliminate xenobiotic compounds from their body without certain gut microbiome species. Few reports mention the digestion of cellulose materials and converting them into β-glucans and β-glucose in the presence of *Verrucomicrobia* bacteria in the gut of the fish. These findings confirmed that they are important for digesting plant cellulose in the fish gut. These aspects have been experimentally proved in carp and further strengthened by reduced cellulase activity in antibiotic-treated fish (van Kessel et al., 2011). Eichmiller et al. (2016) noted that microbes belonging to the Clostridia group produce several bioactive compounds, including propionate, short fatty acid chains, and butyrate, within the host gut tract system to maintain and promote the host’s growth. Identical observations have been made by Baldo et al. (2015) while working on these bacteria, which perform the function of collagen digestion as they produce collagen-degrading enzymes. Several workers have reported that the Fusobacteria present in the gut tract of fish produce vitamin B12, which is vital in performing the function of the growth and development process (Roeselers et al., 2011; Ye et al., 2014; Eichmiller et al., 2016; Liu et al., 2016).

## Advanced tools for the analysis of antimicrobial compounds

The antimicrobial compounds from microbiomes and their functional role in antimicrobial resistance are well understood, however, their large-scale applications in aquaculture farming and other sectors have been hindered due to less investment and the high cost of production of such compounds from natural resources. In addition to this, we also need innovative and sophisticated advanced techniques for their discovery and analysis, particularly from the gut microbiome species of fish and shellfish. Traditional methods of analysis of antimicrobial compounds from microorganisms include several types of diffusion methods. These methods are agar disk diffusion, agar well diffusion, antimicrobial gradient, and agar plug diffusion (Rex et al., 2010; Balouiri et al., 2016). The agar disk diffusion method is the most commonly used test to assess pathogen susceptibility, and in this method, a desired concentration of the compound to be tested is placed on the surface of agar containing microbes. Here, antimicrobial agents enter the test compound, diffuse into the agar, and inhibit the proliferation of susceptible microbes, which can be measured. Though his method cannot accurately determine the minimal inhibitory concentration, it is simple and less expensive (Weinstein et al., 2019). Another method is the antimicrobial gradient, which combines dilution and diffusion to determine the inhibitory concentration value of antibiotics and antimicrobial activity, including antifungal. The microbe’s cell damage and viability and its antimicrobial resistance capacity can be determined by the flow cytofluorometric protocol (Weinstein et al., 2019; Landaburu et al., 2020). Therefore, in summary, traditional protocols for determining antimicrobial resistance of the microbes have been replaced in the recent past by more advanced tools like bioinformatics-based subtractive genomics and metabolic pathway analyses. (Uddin et al., 2015; Zhan et al., 2016; Khan et al., 2020; Shahid et al., 2020). However, the use of protocols based on in silico approaches is more advantageous in the future, but the full knowledge of their capabilities has not been explored yet. Nonetheless, other molecular and genomic technologies have recently seen some success.

Several workers have mentioned earlier that infection-based antimicrobial activity has enhanced the development of identification of promising therapeutics protocols, in the management of microbial diseases (Hackbarth et al., 2002), as well as in the investigation of antibacterial inhibitors like peptide deformylase, which is a vital ingredient in the survival of pathogenic strains, such as *Mycobacterium smegmatis* (Chen et al., 2004; Teo et al., 2006; Kaplan et al., 2012; Naor et al., 2019). In recent years, Fanelli et al. (2020) mentioned that due to the emergence of molecular tools, notably genomics, transcriptomics, and proteomics, has gained momentum in the development of bioinformatics knowledge to identify novel drugs and several other lead bioactive compounds that have the properties of antimicrobials. Further, it is also mentioned that genome mining technologies can be used to detect and analyze the biosynthetic gene clusters of such antimicrobial compounds. Vamathevan et al. (2019) emphasized that artificial intelligence and machine learning technologies also allow scientists to develop alternate protocols to battle against antimicrobial resistance using microbiome ingredients.

## Challenges and future prospects

Several efforts are being made to replace antibiotic drugs and chemicals with natural products without diluting the impact of preventing bacterial and viral infectious diseases in fish and shellfish for the development of sustainable aquaculture all over the world. Many have suggested the use of plant-based phytochemical compounds, and there are reports on the use of marine bioactive compounds derived from various sources of animals and microorganisms. However, all these products derived from natural resources are still experimental stage and may take a long time to become a reality. Research investigations on the use of bioactive compounds derived from gut microbiomes of fish and shellfish for preventing infectious diseases in aquaculture farming have gained momentum in the recent past and several products like probiotics, prebiotics, synbiotics, bacteriophage technology, etc., are now available in the market and are being used in the aquaculture farming. However, using these products has its limitations, with variable results on the growth and performance of cultured organisms. Some antimicrobial compounds like bacteriocins, sebastenoic acid, spartanamycin, anthramycin, and turbinmicin, which have been discovered in microbiomes and experimentally proved to have great potential to replace antibiotic drugs, need further research investigations. The genome mapping, transcriptome studies, and metagenomics studies of such antimicrobial-producing microbiome species are essential and to be done on a priority basis. The number of novel bioactive compounds mentioned in this review illustrates the importance of the antibiotic properties of the gut microbiome species and some innovative ways the search for new antimicrobials in the management of various infectious diseases. Further investigation of these microbiomes will pave the way for searching for new antimicrobial compounds and present us with potent antimicrobials. With such novel compounds, antimicrobial resistance can be reduced among cultivable species in aquaculture farming. Investigations of such antimicrobial bioactive compounds from the microbiome communities will unlock a new innovative concept in animal health management and aquaculture farming, with effects on cost efficiency, species health, productivity, and yield enhancement. Therefore, introducing such new methods for therapeutic purposes in the treatment of bacterial diseases in cultivable organisms will be highly beneficial to the aquaculture industry.

Another challenge in investigating antimicrobial compounds from microbial communities in the marine environment includes the mass culture of the microbes and the expression of genes responsible for producing antimicrobial compounds under in vitro conditions. Because under variable environmental conditions, there is a possibility of changing the structural profile of microbiomes and their metabolites. Several ecological factors significantly disturb microbial diversity and the bioactive compounds/metabolites they produce. It has been suggested that the use of some selective and targeted media for the isolation of bioactive marine Actinobacteria from fish needs further investigation. It has also been observed that the presence of specific signaling molecules found in the bacteria inhabiting their natural environment has a direct impact on the growth of some marine bacterial flora. Advanced molecular technologies, such as next-generation sequencing, metagenomics, metabolomics, and genome mining, are more frequently being used to assess in-depth knowledge of microbial communities. In the recent past, many studies have emphasized the use of metagenomic sequencing and bioinformatic tools to characterize the functionality of microbial communities, antimicrobial resistance genes, and bioactive metabolite genes in marine organisms, including those from rarer deep-sea fish species.
